# Dual targeted therapy in patients with psoriatic arthritis and spondyloarthritis: a real-world multicenter experience from Spain

**DOI:** 10.3389/fimmu.2023.1283251

**Published:** 2023-10-23

**Authors:** Cristina Valero-Martínez, Judit Font Urgelles, Meritxell Sallés, Beatriz E. Joven-Ibáñez, Alexia de Juanes, Julio Ramírez, Xavier Juanola, Raquel Almodóvar, Ana Laiz, Mireia Moreno, Manel Pujol, Emma Beltrán, José Antonio Pinto-Tasende, Laura Crespí, Luis Sala-Icardo, Santos Castañeda, Rosario García-Vicuña

**Affiliations:** ^1^Rheumatology Unit, Hospital U. de La Princesa, IIS-Princesa, Madrid, Spain; ^2^Rheumatology Unit, Hospital U. Germans Trias i Pujol, Badalona, Spain; ^3^Rheumatology Unit, Althaia Xarxa Assistencial Universitària, Manresa, Spain; ^4^Rheumatology Department, Hospital U. 12 Octubre, Madrid, Spain; ^5^Rheumatology Unit, Hospital Clinic, Barcelona, Spain; ^6^Rheumatology Unit, Hospital U. Bellvitge, Hospitalet de Llobregat, Barcelona, Spain; ^7^Rheumatology Unit, Hospital U. Fundación Alcorcón, Madrid, Spain; ^8^Rheumatology Unit, Hospital de la Santa Creu i Sant Pau, Barcelona, Spain; ^9^Rheumatology Unit, Hospital U. Parc Taulí, I3PT (UAB), Sabadell, Spain; ^10^Rheumatology Unit, Hospital U, Mútua Terrassa, Barcelona, Spain; ^11^Rheumatology Unit, Hospital del Mar, Barcelona, Spain; ^12^Rheumatology Unit, Complexo Hospitalario U. de A Coruña, A Coruña, Spain; ^13^Rheumatology Unit, Hospital de Manacor, Manacor, Spain; ^14^Rheumatology Unit, Hospital de Torrejón de Ardoz, Madrid, Spain; ^15^Cátedra Universidad Autónoma de Madrid UAM-Roche, enfermedad pulmonar intersticial difusa (EPID)-Future, Department of Medicine, Universidad Autónoma de Madrid (UAM), Madrid, Spain; ^16^Department of Medicine, Universidad Autónoma de Madrid (UAM), Madrid, Spain

**Keywords:** biologics, spondyloarthritis, combination (combined) therapy, psoriatic arthritis (PsA), inflammatory bowel disease, safety, real word data, multicenter study

## Abstract

Dual targeted therapy (DTT) has emerged as a promising approach in patients with refractory spondyloarthritis (SpA) or psoriatic arthritis (PsA) and extra-musculoskeletal manifestations of both diseases, but its effectiveness/safety ratio still remains unclear. This is a retrospective, real-world multicenter study in refractory SpA and PsA patients with simultaneous use of two biological or synthetic targeted agents. Effectiveness was assessed using Ankylosing Spondylitis Disease Activity Score with C-reactive protein (ASDAS-CRP) and Disease Activity in Psoriatic Arthritis (DAPSA) Score. We identified 39 different DTT combinations in 36 patients (22 SpA; 14 PsA), 25 of them with concomitant inflammatory bowel disease. The most commonly used combinations were TNF inhibitor plus antagonist of the IL12/23 pathway, followed by TNF inhibitor plus IL-17 antagonist. During a median exposure of 14.86 months (IQR 8-20.2), DTT retention rate was 69.4% (n=25/36; 19 SpA, 6 PsA). Major clinical improvement (change in ASDAS-CRP > 2 or improvement > 85% in DAPSA) was achieved in 69.4% of patients (n=25/36 therapeutical combinations; 17/21 SpA, 8/15 PsA), with a 58.3% (n=21/36 combinations; 15/20 SpA, 6/13 PsA) low-activity/remission rate. Of the patients who were receiving glucocorticoids, 55% managed to withdraw them during follow-up. Interestingly, only four serious adverse events in three patients were observed, leading to DTT discontinuation.

## 1 Introduction

Combination therapy with either biologics (b) or targeted synthetic (ts) disease−modifying antirheumatic drugs (DMARDs) and conventional synthetic (cs) DMARDs has become an accepted practice in some difficult-to-treat patients with psoriatic arthritis (PsA) and spondyloarthritis (SpA), according to the predominant involvement of peripheral, or extra-musculoskeletal (extra-MSK) domains (1, 2). In patients with refractory disease, combination therapy involving bDMARDs/tsDMARDs has been proposed as an alternative approach to mitigate the risk of “escape mechanisms” that can result in a loss of response to bDMARDs (3). Therefore, this therapeutic strategy may provide synergistic benefits by targeting two different pathogenic pathways implicated in those diseases.

However, the use of two b/tsDMARDs in combination is usually not recommended in clinical practice or guidelines of immune-mediated diseases, due to lack of consistent evidence, potential safety concerns, and high cost. The potential adverse events (AEs) of dual blockade of different inflammatory pathways are still poorly studied. Conceivably, the rate of unexpected AEs, and especially the risk of infections could be increased due to a double immunosuppression mechanism derived from some combinations. To date, some reports have summarized the elevated safety risk associated with certain biological combinations, particularly in the context of rheumatoid arthritis (RA) (4, 5).

Despite those concerns, dual targeted therapy (DTT) is an emerging research topic in several fields of medicine following the successful experiences reported in refractory patients with inflammatory bowel disease (IBD) (6–9).

However, to date, little research has been published about DTT in rheumatic diseases, regarding appropriate combinations or target patients who could benefit more (4, 5). Previous case-series in PsA showed favorable efficacy results with DTT although some patients exhibited AEs (10, 11). In contrast, our previous case series with DTT in a cohort of nine selected patients with refractory multidomain SpA showed encouraging results (12). Herein, our aim was to assess the real-world experience of DTT in an extended multicentric cohort of refractory patients with PsA and SpA.

## 2 Methods

This is an observational, retrospective, multicenter, cross-sectional study conducted in Spain. We enrolled PsA and SpA patients exposed to simultaneous (combined) use of two biological or synthetic targeted agents with different therapeutic targets, from April 2017 to December 2022. SpA patients fulfilled axial or peripheral ASAS criteria for SpA (13) and PsA patients fulfilled the classification criteria for psoriatic arthritis (CASPAR) criteria (14). Sociodemographic, clinical, laboratory, and safety data were collected from electronic medical records.

We define dual or combined therapy as the simultaneous use of two targeted therapies in the same patient and at the same time. In particular, in our series, three patients out of 36 received two different combinations of DTT in different moments of their evolution. Combinations including apremilast were only identified in two patients, with insufficient data to assess properly efficacy or safety, and therefore, we did not include this agent in the present series.

As the outcomes for effectiveness were focused on the rheumatologic domains and considering the recognized differences between SpA and PsA in some pathogenic pathways, clinical presentation, and response to treatments, we choose to address both entities separately, that also warrant the use of distinctive disease activity indexes: Ankylosing Spondylitis Disease Activity Score with C-reactive protein (ASDAS-CRP) and Disease Activity in Psoriatic Arthritis (DAPSA) Score. The cut-off points for remission/low activity criteria were ASDAS-CRP <1.3/<2.1, and DAPSA <4/<14, respectively. Major clinical improvement (MCI) was defined as a change in ASDAS-CRP >2 or improvement greater than 85% in DAPSA. Data analysis included descriptive statistics for categorical and continuous variables and was performed using SPSS 20.0 software.

This study complies with the principles of the Declaration of Helsinki, and the locally appointed research ethics committee of the Hospital Universitario de la Princesa has approved the research protocol (reference number 5177). This is a non-intervention study and patient data was anonymized in the databases provided to all centers, guaranteeing the confidentiality of personal information. All patients received a patient information sheet about the study and provided written informed consent for the off-label use of the dual biologic therapy, in accordance with standard clinical practice.

## 3 Results

A total of 39 DTT combinations (23 SpA, 16 PsA) were identified in 36 patients (22 SpA, 14 PsA), 69.4% of them (25/36) presented concomitant IBD (20 SpA, 5 PsA). The main characteristics and outcomes of SpA and PsA patients on DTT are summarized in **Tables 1**, **2**. The type and indications for different combinations are shown in **Table 3**. The main indication for initiation of DTT was active musculoskeletal (MSK) disease, upon approval of the corresponding specialists if IBD, psoriasis (Ps) or uveitis were under control monotherapy. In 10 patients, 11 combinations for a double indication MSK plus extra-MSK symptoms, were agreed with gastroenterologists (10 IBD) or dermatologists (1 Ps). In 4 patients, the recommendations for 3 isolated active IBD or 1 Ps were provided by the corresponding specialists. Almost all patients presented moderate-high MSK activity at baseline (33/36 patients). Patients with PsA had received a higher number of bDMARDs/tsDMARDs prior DTT compared to those with SpA (median 5 ± 3 vs 3 ± 2).

**Table 1:** Main clinical features and outcomes of PsA patients under DTT combination.

Age-gender	Disease phenotype +- extra-MSK disease (duration MSK of disease)	Previous bDMARDs/tsDMARDs	Indication for DTT	DTT	DTT exposure (months)	Disease activity at 6 months*	Disease activity at last evaluation*	MI	Permanent withdrawal of DTT (reason)
Case 1 62-year-old female	Peripheral and axial (40 years)	IFX, ETN, GOL, SEC	MSK disease	SEC + ETN	8	Moderate	Low	Yes	Yes (AEs)
Case 2 61-year-old female	Peripheral and axial (9 years)	IFX, ADA, ETN, CTZ, SEC, IXE, TOF	MSK disease	SEC + GOL	26	Low	Low	Yes	No
Case 3 64-year-old male	Peripheral (20 years)	IFX, ADA, ETN, GOL, SEC, IXE	MSK disease	SEC + ADA	6	Moderate	High	No	Yes (inefficacy: MSK activity)
Case 4 66-year-old female	Peripheral (19 years)	IFX, ADA, IXE	MSK disease	IXE + ADA	9	Moderate	Moderate	Yes	Yes (inefficacy: MSK activity)
Case 5 49-year-old female	Peripheral and axial + uveitis (21 years)	ADA, IFX, CTZ, GOL, IXE, UPA	MSK disease	IXE + GUS	9	Moderate	Moderate	Yes	No
Case 6 34-year-old female	Peripheral (23 years)	IFX, ADA, ETN, CTZ, GOL, SEC, UST	Psoriatic disease	SEC + ETN	12	**	**	**	No
Case 7 70-year-old male	Axial + IBD (19 years)	IFX	IBD + MSK disease	UST + ADA	12	High	High	No	Yes (inefficacy: MSK and IBD activity)
Case 8 58-year-old female	Peripheral and axial + IBD (9 years)	ETN, ADA, CTZ, UST, SEC, VED	IBD + MSK disease	VED + GUS	5	Not applicable	High	No	Yes (inefficacy: IBD activity)
			IBD	VED + UST	14	High	High	No	Yes (inefficacy: MSK and IBD)
Case 9 54-year-old male	Peripheral and axial (7 years)	ADA, IFX, SEC, UST, GUS	MSK + psoriatic disease	GUS + ABT	21	Moderate	Low	Yes	Yes (patient decision)
Case 10 70-year-old male	Axial + IBD (19 years)	IFX	IBD + MSK disease	VED + ADA	3	High	High	No	Yes (inefficacy: MSK and IBD activity)
Case 11 32-year-old female	Peripheral and axial + IBD (4 years)	IFX, UST	MSK disease	UST + ADA	2	Not applicable	Not applicable	No	Yes (pregnancy)
			MSK disease	UST + CTZ	5	High	Not applicable	No	Yes (inefficacy: MSK activity)
Case 12 37-year-old female	Peripheral and axial + IBD (22 years)	ADA, IFX, UST	MSK disease	UST + CTZ	19	Low	Low	Yes	No
Case 13 44-year-old female	Peripheral (12 years)	ADA, ETN, IFX, SEC, IXE, UST, GUS,	MSK disease	GUS + CTZ	9	Low	Low	Yes	No
Case 14 40-year-old female	Peripheral (20 years)	ADA, ETN, IFX, GOL, IXE, SEC, UST, TOF	MSK disease	GOL + BRODA	8	Low	Low	Yes	No

ABT, abatacept; ADA, adalimumab; AEs, adverse events; b/tsDMARDs, biologic or targeted disease-modifying anti-rheumatic drugs; BRODA, brodalumab; CTZ, certolizumab pegol; DTT, dual targeted therapy; ETN, etanercept; GOL, golimumab; GUS, guselkumab; IBD, inflammatory bowel disease; IFX, infliximab; IXE, ixekizumab; MI, major improvement; MSK, musculoskeletal; PsA, psoriatic arthritis; SEC, secukinumab, TCZ, tocilizumab; TOF, tofacitinib; VED, vedolizumab; UST, ustekinumab.

*PsA disease activity was measured by DAPSA or ASDAS-CRP in peripheral or axial involvement, respectively.

**This patient showed high psoriatic activity but low MSK activity at start of DTT, therefore the efficacy of DTT in MSK domain was not analyzed.

**Table 2:** Main clinical features and outcomes of SpA patients under DTT combination.

Age-gender	Disease phenotype +- extra-MSK disease (duration of MSK disease)	Previous bDMARDs/tsDMARDs	Indication for DTT	DTT	DTT exposure (months)	Disease activity at 6 months*	Disease activity at last evaluation*	MI	Permanent withdrawal of DTT (reason)
Case 1 28-year-old male	Peripheral and axial + uveitis (22 years)	IFX, ADA, ETN, CTZ, GOL, SEC, TCZ	MSK disease	SEC + GOL	68	Low	Remission	Yes	No
Case 2 46-year-old male	Peripheral and axial + uveitis (34 years)	ADA, ETN, GOL, CTZ, SEC	MSK disease	ETN + SEC	38	Low	Remission	Yes	No
Case 3 32-year-old male	Peripheral and axial + IBD (24 years)	IFX, ADA, ETN, GOL	MSK disease	GOL + RIS	24	Remission	Remission	Yes	No
Case 4 22-year-old male	Peripheral + IBD (20 years)	IFX, ADA, ETN, UST, VED	MSK disease	VED + GOL	20	Remission	Remission	Yes	No
Case 5 75-year-old male	Peripheral + IBD (3 years)	IFX, ADA, UTK	MSK disease	UST + GOL	18	Low	Low	Yes	No
Case 6 60 year-old female	Peripheral and axial + IBD (7 years)	IFX, ADA, ETN, SEC, UST	IBD + MSK disease	GOL + UST	17	High	Low	Yes	No
Case 7 40-year-old male	Peripheral and axial + uveitis + IBD (22 years)	IFX, ADA, CTZ	IBD + MSK disease	UST + GOL	1	Not applicable	Not applicable	No	Yes (AEs)
			IBD + MSK disease	UST + ETN	41	High	High	Yes	No
Case 8 41 year-old female	Axial + IBD (9 years)	CTZ	IBD	CTZ + UST	15	**	**	**	Yes (inefficacy: IBD activity)
Case 9 25 year-old male	Peripheral + uveitis + IBD (23 years)	IFX, ADA, ETN, TOF, TCZ	IBD + MSK disease	IFX + TOF	22	Remission	Low	Yes	No
Case 10 41 year-old male	Axial + IBD (4 years)	IFX, UST	MSK disease	ADA + UST	33	Low	Remission	Yes	No
Case 11 61 year-old female	Peripheral + axial + IBD (17 years)	UST	MSK disease	ADA + UST	17	Low	High	Yes	No
Case 12 38 year-old male	Axial + IBD (10 years)	ADA	IBD	IFX + UST	16	Low	Low	Yes	No
Case 13 49 year-old male	Peripheral + uveitis + IBD (23 years)	IFX, CTZ, ETN	IBD + MSK disease	ADA + UST	37	Remission	Remission	Yes	No
Case 14: 41 year-old male	Axial + IBD (13 years)	IFX, ADA, CTZ, UST	MSK disease	UST + ADA	13	High	High	No	Yes (inefficacy: IBD and MSK activity)
Case 15: 36 year-old male	Peripheral + IBD (4 years)	IFX	IBD + MSK disease	IFX + UST	16	High	High	No	No
Case 16: 63 year-old male	Peripheral and axial + IBD (24 years)	ADA, IFX, UST	MSK disease	UST + CTZ	2	Not applicable	Low	Yes	No
Case 17: 55 year-old female	Peripheral and axial + IBD (7 years)	IFX, ADA, ETN, CTZ, UST	MSK disease	UST + CTZ	13	Remission	Remission	Yes	No
Case 18: 56 year-old female	Axial + IBD (19 years)	ADA	IBD	ADA + UST	14	**	**	**	No
Case 19: 50 year-old female	Peripheral and axial + IBD (2 years)	ADA, IFX	IBD + MSK disease	ADA + UST	26	Low	Low	Yes	No
Case 20 50 year-old male	Peripheral and axial + IBD (1 year)	ADA, UST	MSK disease	CTZ + UST	8	Low	Low	Yes	No
Case 21 47 year-old female	Peripheral + IBD (13 years)	ADA, IFX, UST	MSK disease	ADA + UST	12	Remission	Remission	Yes	No
Case 22 50 year-old male	Axial + IBD (12 years)	ADA, IFX, UPA, VED, UST	IBD + MSK disease	ADA + VED	5	Not applicable	High	No	Yes (AEs)

ADA, adalimumab; AEs, adverse events; b/tsDMARDs, biologic or targeted disease-modifying anti-rheumatic drugs; CTZ, certolizumab pegol; DTT, dual targeted therapy; ETN, etanercept; GOL, golimumab; IBD, inflammatory bowel disease; IFX, infliximab; IXE, ixekizumab; MI, major improvement; MSK, musculoskeletal; RIS, risankizumab; SEC, secukinumab; SpA, Spondyloarthritis; SpA, spondyloarthritis; TCZ, tocilizumab; TOF, tofacitinib; VED, vedolizumab; UPA, upadacitinib; UST, ustekinumab.

* SpA disease activity was measured by ASDAS-CRP.

**These patients showed low SpA MSK activity at the start of DTT, therefore the efficacy of DTT in the MSK domain was not tested.

**Table 3:** Main indications and effectiveness across the different combinations clustered by classes of targeted therapies used.

	Spondyloarthritis (n= 23, 22 patients)							Psoriatic artrhitis (N= 16, 14 patients)								
Combinations (classes)	All indications (n=23)	Active MSK (n=13)	Active MSK + IBD (n=7)	Active IBD (n=3)	Naive to 1 class*	Naive to 1 drug#	MCI (n, %)	All Indications (n=16)	Active MSK (n=10)	Active MSK + IBD (n=3)	Active IBD (n=1)	Active MSK + skin Ps (n=1)	Active skin Ps (n=1)	Naive to 1 class*	Naive to 1 drug#	MCI (n,%)
Anti TNF+ anti IL-12/23	17	8	6	3**	7	14	11 (73,3)	4	3	1				0	4	2 (50)
Anti TNF + anti IL-17	2	2			0	0	2 (100)	6	5				1***	0	2	4 (80)
Anti TNF + anti IL-23	1	1			1	1	1 (100)	1	1					1	1	1 (100)
Anti TNF + vedolizumab	2	2			0	1	1 (50)	1		1				1	1	1 (100)
Anti TNF + JAKi (Tofacitinib)	1		1		0	0	1 (100)									
Anti IL17 + anti IL-23								1	1					1	1	1 (100)
Anti IL-23 + ABT								1				1		1	1	1 (100)
Vedolizumab + anti IL-12/23								1			1			0	0	0
Vedolizumab + anti IL-23								1	0	1				1	1	0

*Refers to a class of targeted therapy that has not been previously used in monotherapy;

#Refers to a drug that has not been previously use in monotherapy, often into the same class of the targeted therapy used in combination

**2/3 patients had low MSK activity at the onset of combinations and therefore, were not included in the assesment of MSK MCI.

*** The patient has no MSK activity at the onset of combinations and therefore, were not included in the assesment of MSK MCI.

ABT, Abatacept; IBD, inflammatory bowel disease; JAKi, JAK inhibitor; MCI, major clinical improvement; MSK, musculoskeletal; Ps, psoriasis.

Monotherapy with at least one of the two therapies used in the combination had previously failed in most patients (n=32; 88%) In 27/39 (70%) combinations (11 PsA, 16 SpA) the patient was naïve to one of the two drugs in combination, but in 15/23 SpA (65%) and 11/16 PsA (69%) DTT, the patients had been previously exposed to both classes of targeted therapies used in combination (**Table 3**). While some patients had not tried all available therapeutic targets for their conditions when DTT was initiated, 4 patients incorporated a new medication through compassionate off-label use (2 guselkumab, 1 brodalumab, 1 risankizumab) before approval of these drugs for PsA or SpA in our country.

Nineteen different combinations were found (**Tables 1**, **2**), and the most common class combination was a TNF inhibitor (anti-TNF) plus either an IL12/23 antagonist (anti-IL12/23) (n=22; 56.4%) or an IL17 inhibitor (anti-IL17) (n=9; 23%) (See **Table 3** for detailed combined classes and indications). In IBD patients, five therapeutical combinations included vedolizumab (VED) and just one combination included an oral JAK inhibitor (JAKi). The most frequent drugs used in dual regimens were ustekinumab (UST) combined with adalimumab or certolizumab.

The median exposure to DTT was 14.86 months (IQR 8-20.2). At the end of follow-up, the retention rate of patients with DTT was 69.4% (25/36; 19 SpA; 6 PsA). Fourteen DTT combinations (35,8%) were discontinued during follow-up: 9/39 (22,2%) due to uncontrolled disease, 3/39 (7,6%) due to AEs, and 2/39 due to other causes (pregnancy and patient decision).

**Figure 1:**
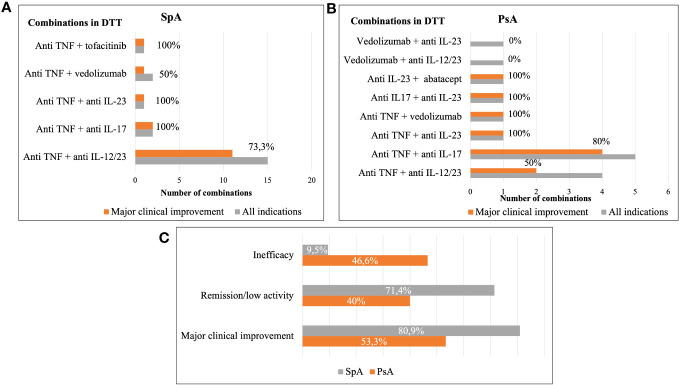
Main effectiveness of dual targeted therapy. Results are shown as percentage of patients achieving different outcomes by type of combinations clustered by classes of drugs used in DTT in SpA **(A)**, PsA **(B)** and in the overall population **(C)**. DTT, dual targeted therapy; SpA, spondyloarthritis; PsA, psoriatic arthritis.

Regarding clinical efficacy in MSK disease, we analyzed only 36 combinations (21 SpA, 15 PsA) in 33 patients (20 SpA, 13 PsA) who exhibited high or moderate MSK activity at baseline. Of 30/36 combinations that reached 6 months of follow-up, 60% (18/30: 14/21 SpA, 4/15 PsA) achieved remission or low MSK activity. During complete follow-up, the overall remission/low activity rate across all combinations was 58.3% (21/36; 15/21 SpA, 6/15 PsA), and 69.4% (25/36 combinations; 17/21 SpA, 8/15 PsA) reached MCI at some point during follow-up (**Table 3**; **Figure 1**).

From 25 patients who were naïve to one of the two drugs in combination and exhibited MSK activity at baseline, 19 (76%) demonstrated MCI (6/11 PsA, 13/14 SpA). Conversely, 54% (6/11) of patients previously exposed to the tested drugs in DTT achieved MCI (1/4 PsA, 5/7 SpA). Of 20/36 patients who were under glucocorticoid therapy at baseline, 55% (11/20: 6 SpA, 5 PsA) were able to discontinue them during follow-up.

Only four serious AEs (SAEs) were identified in three patients leading to DTT discontinuation. In PsA cases, a cirrhotic woman with multiple comorbidities under secukinumab plus etanercept developed staphylococcal bacteremia at 8 months (**Table 1**: case 1). In SpA cases, a man under golimumab and UST presented a non-infectious acute hypersensitivity pneumonitis in the first month (**Table 2**: case 7) and another one under adalimumab and VED presented cytomegalovirus colitis and esophageal candidiasis (**Table 2**: case 22). Another two patients discontinued DTT, due to pregnancy and patient decision, respectively.

## 4 Discussion

DTT has been proposed as a feasible approach in selected patients with rheumatic diseases and a history of failure to multiple drugs, or refractory extra-MSK symptoms. Use of DTT, in PsA or SpA aims at improving the clinical efficacy of previous biologics used in monotherapy for MSK manifestations, or as an add-on therapy for uncontrolled or new onset extra-MSK condition, or to treat *de novo* MSK symptoms in a well-controlled extra-MSK disease under biological monotherapy. Moreover, may be DTT could be integrated into the treat-to-target strategy in severe PsA patients, as achieving sustained minimal disease activity requires a rigorous approach of all disease domains (15).

Herein, we present a multicenter real-world experience on 39 DTT, combinations in 22 SpA and 14 PsA patients, 69% with concomitant IBD. The most frequent indication for DTT was the presence of MSK symptoms. Our patients achieved drug retention in more than half of the cases with a significant reduction in glucocorticoids and a median exposure of more than one year. In those patients who presented MSK activity at baseline, 69.4% of the combinations achieved MCI at some point during the follow-up, despite being multi-refractory patients in many cases.

Our data on DTT, are comparable to those described in other studies on IBD patients on DTT (6–9). The most common drug combinations in these studies were VED in combination with anti-TNF, tofacitinib or anti-IL12/23 therapy, or anti-TNF in combination with anti-IL-12/23, with heterogeneity of effectiveness results between combinations (6–9). Alayo et al. (7) observed a greater clinical remission rate with the combination of anti-IL12/23 plus anti-TNF. In a recent Finnish multicenter study (8), the most successful combination was adalimumab plus UST, and no SAEs were reported. In our case series, DTT was indicated for IBD or IBD+MSK activity in 14/25 patients with associated IBD with at least five different combinations, which prevent us from drawing conclusions about the best combination.

Regarding safety, in two meta-analysis (7, 8) in patients diagnosed with IBD who were on DTT, the pooled rates of SAEs were 9.6% and 6.5%, respectively, and the most common SAEs reported were infections. None of the combination therapies assessed in these studies revealed any new safety concerns. Our data, with a 10% SAEs, all infections, are in line with those results and consistent with those AEs previously described for the same drugs in monotherapy, but a rigorous comparison should include adjusted incidence rates. Besides, safety results in IBD should be interpreted with caution, given the better safety profile of UST or VED in monotherapy compared to other drugs (3, 16), since it is one of the most repeated combinations in these studies.

In contrast with a growing real-world data, only one phase II randomized controlled trial (RCT) on combination therapy with biologics in IBD have been published yet (the VEGA study) yet (17). This study evaluated the combination therapy with guselkumab plus golimumab vs. both drugs in monotherapy in patients with ulcerative colitis. This study reported equivalent AEs rate for the three treatment groups. After 12 weeks, dual therapy showed better clinical response than both drugs alone, with significant remission rates compared to monotherapy arms. Another phase IV clinical trial (EXPLORER) is currently underway in patients with Crohn’s disease, combining adalimumab, VED, and methotrexate (NCT02764762, clinicaltrials.gov).

Data on DTT in rheumatic diseases are currently limited, with most studies conducted in RA (4, 5). Two older RCT that evaluated the combination of etanercept plus anakinra or abatacept (18, 19), showed no treatment benefit of the combination therapy over monotherapy with increased risk of AEs, including higher rates of infections with combination therapy (18, 19). Other studies, including a RCT (20), have explored the combination of rituximab plus anti-TNF, showing an improvement in efficacy without more SAEs notification compared to rituximab in monotherapy (20, 21). Another clinical trial showed a rapid decrease in disease activity in RA patients treated with bimekizumab plus certolizumab compared with certolizumab plus placebo. They observed a higher number of non-serious infections in the dual treatment group with the same number of serious infections between both groups (22). A case series in six refractory patients (five RA and one PsA) explored combination treatment with tofacitinib and other biologic agents (tocilizumab, rituximab, and etanercept), and no patient experienced SAEs (23). However, comparisons between SpA or PsA and RA populations should be avoided, given the well-known increased risk in RA of serious infections, older age, wider corticosteroid use, and comorbidities.

In PsA, unlike RA, there are no published RCT on the efficacy and safety of two different targeted drugs in combination, and the available information is limited to observational studies. Several case series have explored DTT for the treatment of refractory PsA with effective results in several patients with anti-IL12/23 (11, 24–27) or anti-IL23 (27–30) in combination with an anti-TNF. The most used combination was anti-IL12/23 with anti-TNF which results effective in 15/18 patients and AEs were shown in 9/18 patients (7 of them were infections and 4 were SAEs). Six patients discontinued DTT due to AEs (11, 23–30). In our study, 2/4 PsA patients under this combination discontinued DTT due to inefficacy, but no SAE were recorded. Other reports have also documented positive efficacy outcomes in three patients with PsA under anti-IL23 + anti-TNF without any adverse events (27–30). Our experience in two patients with this combination (1 PsA, 1 SpA) was also successful and one additional combination with anti-IL23 plus IL17 in a PsA also rendered positive results.

In our patients with PsA, the most commonly prescribed DTT was anti-IL17 plus anti-TNF with a high efficacy rate (80% MCI) which suggests that this combination deserves more research as we have found only a case report on this DTT, which proved unsuccessful due to psoriasis activity (27). A novel bispecific monoclonal antibody (ABT-122) targeting TNF and IL-17A has demonstrated, in patients with PsA, acceptable tolerability compared to adalimumab monotherapy during a phase II trial (31), but combination therapy did not add an additional efficacy benefit compared to monotherapy. A recent case series described six patients with PsA treated with tofacitinib plus anti-IL23 (2/6), anti-IL12/23 (1/6) or anti-IL17 (3/6), reporting disease improvement in all patients and no SAEs were reported (32).

Another alternative for patients with refractory PsA is the combination of apremilast and biological therapy, since apremilast has a good safety profile even in combination (33, 34), but the presumed risk of infections is not comparable with the combination of other biological agents. Of note, a controlled clinical trial (AFFINITY) is ongoing to evaluate the efficacy of guselkumab plus golimumab combination treatment in patients with PsA and inadequate response to prior anti-TNF therapies compared with guselkumab monotherapy (NCT05071664, clinicaltrials.gov).

Little information is available about DTT in SpA and all patients are included in IBD studies, where the available data on SpA activity are generally insufficient (35–49). A recent European multicenter observational study involving patients with IBD and DTT (49), reported the presence of 25 patients with SpA, but did not specify the combinations or outcomes for SpA-IBD. Most frequent combinations included anti -TNF plus VED, followed by anti-TNF or VED plus UST or other “IL inhibitors”. Although findings suggest DTT can be a promising strategy, description of the results for overall extraintestinal manifestations makes comparisons with ours difficult. Additionally, they draw attention to the risk of serious or opportunistic infections, with a non-adjusted rate similar to ours and other studies (7, 8).

In studies where accuracy data on SpA were available (35–48), we identified a total of 27 combinations for IBD associated SpA, mostly involving VED with anti-TNF or JAKi, and less frequent combinations of UTK with anti-TNF, VED, or JAKi. In those cases, 25 combinations reported comprehensive efficacy and safety results regarding SpA activity, that were extended to intestinal domains in 18 DTT. No SAEs were reported, although in most cases the follow-up periods were less than one year (35–48).

According to the above data, a recent review highlights how VED or UST are often used as anchor therapies in drug combinations in IBD associated SpA, due to their intestinal selectivity or favorable safety profiles (3), also demonstrated for UST in PsA (16).

In our SpA population, the most frequent drug combination was anti-TNF plus anti-IL12/23 (17/23 combinations) likely selected due to active concomitant IBD indication in 9 patients, which restricted the use of IL17-targeting therapies that have shown highly effective for MSK domains.We obtained comparable efficacy outcomes compared to the previously described cases. Two patients with SpA combined anti-TNF plus anti-IL17A, and to our knowledge, no data on the association between anti-TNF and anti-IL17A inhibitors in SpA have been published yet. Previous research, in rat models of SpA and RA (50, 51), has demonstrated that dual inhibition of TNF and IL17 significantly reduces inflammation and structural damage compared with monotherapy, suggesting a synergic benefit.

Interestingly, our study revealed higher efficacy responses in the SpA group compared to those with PsA (71% vs. 40% remission/low disease activity; 80% vs 53% MCI) throughout the follow-up. We cannot rule out that a larger sample size in SpA compared to PsA cases could have influenced those results. Additionally, patients with PsA had experienced a higher number of bDMARDs/tsDMARDs prior DTT compared to those with SpA (median 5 ± 3 vs 3 ± 2), reflecting a more refractory population. Furthermore, it is noteworthy that 7 patients with PsA and axial involvement received either anti-IL23 or anti-IL12/23 therapy, which have not been proven effective in axial domains and are not recommended for this patient profile (2).

Other case series are exploring the combination of biological therapies in patients with rheumatic diseases but with different indications for biological treatment. Malik et al. presented three patients on dual biologics for rheumatic disease (two RA and other with Crohn´s-associated arthritis) and concomitant asthma, combining mepolizumab or omalizumab with anti-TNF and no SAEs were reported (52). Yıldırım et al. reported a case series in patients with Familial Mediterranean Fever and SpA treated with the combination of anakinra or canakinumab with anti-TNF or anti-IL17 or tocilizumab. All patients achieved remission with some dual therapy combinations and no SAEs were revealed (53). Successful experiences with DTT in patients with PsA and severe atopic dermatitis with secukinumab and dupilumab was also reported, without significant AEs (54). In a case series of 28 patients with rheumatic diseases treated with denosumab and biological therapy, a comparable safety was shown between control group compared with biological monotherapy (55).

Our study has several limitations. The retrospective and uncontrolled design, the limited sample size, the heterogeneity of MSK phenotypes and combination treatments, and a wide range of follow-up periods preclude drawing solid conclusions. Additionally, significant percentage of DTT cases were naïve to one of the two drugs, and these patients showed greater MCI compared with non-naïve patients. It is worth noting that some patients in the series had not tried all available therapeutic targets in SpA or PsA, such as JAKi or anti-IL23 drugs, and DTT should be reserved for patients who have not achieved all existing treatment options. However, in 9 out of 14 PsA patients, axial involvement was present, making anti-IL23 therapy an unsuitable option for these cases. Other cases started DTT, as compassionate therapy before our country approved these new drugs for SpA or PsA indications Another clinical situation we encountered was that some patients had controlled IBD but active MSK disease, so it was decided to maintain the drug that controlled the IBD and add another one in combination to treat the MSK symptoms, rather than replacing it with a different one. Finally, although no new safety signals were identified, the study design, a limited follow-up, and the heterogeneous population preclude drawing conclusions about unexpected AEs.

To the best of our knowledge, our study provides the largest and longest series reported to date on DTT, in patients with SpA and PsA. Furthermore, its multicenter design may also reduce the biases of single-center studies. In conclusion, our preliminary results suggest that DTT might be a good therapeutic alternative in selected cases of multidomain, refractory and difficult-to-treat SpA and PsA, with acceptable safety ratio. However, further controlled studies are needed to examine the long-term safety and efficacy of DTT.

## Data availability statement

The raw data supporting the conclusions of this article will be made available by the authors, without undue reservation.

## Ethics statement

This study was approved by CEIm-Fundación para la Investigación Biomédica, Hospital Universitario de La Princesa, Diego de León 62, Madrid (28006), 91 520 22 00 (ext. 17528), ceim.hlpr@salud.madrid.org. The study was conducted in accordance with the local legislation and institutional requirements. This is a non-intervention study and patient data was anonymized in the databases provided to all centers, guaranteeing the confidentiality of personal information. All patients received a patient information sheet about the study and provided written informed consent to off-label use of dual biological therapy, according to standard clinical practice. The waiver of written informed consent for publication was requested and approved by the ethics committee. As a study involving humans, any potentially identifiable images or data are included in this article. The study complies with the principles of the Declaration of Helsinki, and the locally appointed research ethics committee of the Hospital Universitario de la Princesa has approved the research protocol (reference number 5177).

## Author contributions

CV-M: Conceptualization, Data curation, Formal Analysis, Investigation, Project administration, Writing – original draft, Writing – review & editing. JF: Data curation, Project administration, Validation, Writing – review & editing. MS: Validation, Writing – review & editing. BJ-I: Validation, Writing – review & editing. AdJ: Validation, Writing – review & editing. JR: Writing – review & editing. XJ: Validation, Writing – review & editing. RA: Validation, Writing – review & editing. AL: Validation, Writing – review & editing. MM: Validation, Writing – review & editing. MP: Validation, Writing – review & editing. EB: Validation, Writing – review & editing. JP-T: Validation, Writing – review & editing. LC: Validation, Writing – review & editing. LS-I: Validation, Writing – review & editing. SC: Conceptualization, Methodology, Supervision, Validation, Writing – original draft, Writing – review & editing. RG-V: Conceptualization, Data curation, Methodology, Project administration, Supervision, Validation, Writing – original draft, Writing – review & editing.
